# Does socioeconomic status affect mortality subsequent to hospital admission for community acquired pneumonia among older persons?

**DOI:** 10.1186/1477-5751-4-4

**Published:** 2005-04-08

**Authors:** Linda Vrbova, Muhammad Mamdani, Rahim Moineddin, Liisa Jaakimainen, Ross EG Upshur

**Affiliations:** 1 Department of Public Health Sciences, University of Toronto, McMurrich Building, 12 Queen's Park Crescent W, Toronto, ON, M5S 1A8, Canada; 2 Institute for Clinical Evaluative Sciences, 2075 Bayview Avenue, Toronto, ON, M4N 3M5, Canada; 3 Health Policy Management and Evaluation, University of Toronto, McMurrich Building, 2^nd ^Floor, 12 Queen's Park Crescent West, Toronto, ON, Ma5S 1A8, Canada; 4 Faculty of Pharmacy, University of Toronto, 19 Russell Street, Toronto, ON, M5S 2S2, Canada; 5 Department of Family and Community Medicine, University of Toronto, 256 McCaul Street, 2^nd ^Floor, Toronto, ON, M5T 2W5, Canada; 6 Primary Care Research Unit, Department of Family and Community Medicine, Sunnybrook and Women's College Health Sciences Centre, 2075 Bayview Avenue, Toronto, ON, M4N 3M5, Canada

## Abstract

**Background:**

Low socioeconomic status has been associated with increased morbidity and mortality for various health conditions. The purpose of this study was twofold: to examine the mortality experience of older persons admitted to hospital with community acquired pneumonia and to test the hypothesis of whether an association exists between socioeconomic status and mortality subsequent to hospital admission for community-acquired pneumonia.

**Methods:**

A population based retrospective cohort study was conducted including all older persons patients admitted to Ontario hospitals with community acquired pneumonia between April 1995 and March 2001. The main outcome measures were 30 day and 1 year mortality subsequent to hospital admission for community-acquired pneumonia.

**Results:**

Socioeconomic status for each patient was imputed from median neighbourhood income. Multivariate analyses were undertaken to adjust for age, sex, co-morbid illness, hospital and physician characteristics. The study sample consisted of 60,457 people. Increasing age, male gender and high co-morbidity increased the risk for mortality at 30 days and one year. Female gender and having a family physician as attending physician reduced mortality risk.

The adjusted odds of death after 30-days for the quintiles compared to the lowest income quintile (quintile 1) were 1.02 (95% CI: 0.95–1.09) for quintile 2, 1.04 (95% CI: 0.97–1.12) for quintile 3, 1.01 (95% CI: 0.94–1.08) for quintile 4 and 1.03 (95% CI: 0.96–1.12) for the highest income quintile (quintile 5). For 1 year mortality, compared to the lowest income quintile the adjusted odds ratios were 1.01 (95% CI: 0.96–1.06) for quintile 2, 0.99 (95% CI: 0.94–1.04) for quintile 3, 0.99 (95% CI: 0.93–1.05) for quintile 4 and 1.03 (95% CI: 0.97–1.10) for the highest income quintile.

**Conclusion:**

Socioeconomic status is not associated with mortality in the older persons from community-acquired pneumonia in Ontario, Canada.

## Introduction

Community-acquired pneumonia (CAP) is a substantial cause of mortality, morbidity, and health services utilization in the older persons [1]. In Canada pneumonia and influenza are, together, the leading cause of death from infectious disease and sixth leading cause of death overall. In Canada, the annual hospitalization for pneumonia and influenza is 1,358 per 100,000, and in Ontario 1,283 per 100,000 [2]. The high morbidity and mortality associated with CAP makes understanding its epidemiology a research priority.

Current health research increasingly recognizes the existence and contribution of broader determinants of health in explaining differences in health status and health outcomes among populations. Socioeconomic status is an important influence on morbidity and mortality [3,4]. Access to large databases in Canada has allowed for the examination of the relationship of socioeconomic status to specific health outcomes. Recently, Canadian studies have revealed that those with lower socioeconomic status experience higher mortality and morbidity after myocardial infarction [5] and stroke [6]. Such mortality gradients are problematic in a publicly funded health care system, indicating potential problems with unequal access to care. There are few published studies investigating the relation between socioeconomic factors and pneumonia. Previous studies of the association between SES and pneumonia have looked at different endpoints of pneumonia, and used various SES measures, yielding conflicting results.

There is no consensus as to which factors contribute the most to increasing mortality risk from pneumonia, notably whether it is age or co-morbidity that is the deciding variable [7-9]. No studies to date have examined the independent effects of age, gender, co-morbidity and SES on mortality after CAP. This study examines the mortality experience hypothesis of whether there is an association between socioeconomic status and mortality after community-acquired pneumonia in older persons in Ontario, Canada, controlling for age, gender, co-morbidity and other factors.

## Methods

### Study Design and Data Sources

A cohort of patients diagnosed with pneumonia in Ontario hospitals were assembled for 6 years, from April 1, 1995 to March 31, 2001. The inclusion criteria of the cohort were: "most responsible" diagnosis of pneumonia and influenza (codes 480–487 of the International Classification of Diseases, 9^th ^Revision, Clinical Modification [ICD-9-CM][10], age greater than 65 and less than 105 and resident of Ontario. Unpublished data indicates that influenza codes (487) are infrequently used and account for less than .05% of the sample. The most frequently used codes are 485 and 486 which are for pneumonia with no specific isolated causative organism

In order to rule out readmissions, patients who were admitted for pneumonia in the previous 12 months were excluded. Furthermore, in order to focus solely on community-acquired pneumonia, patients transferred from another health-care institution or long-term facility were also excluded.

Hospital discharge abstracts were drawn from the Canadian Institute of Health Information (CIHI) database. The abstracts contained information pertaining to the index admission, age and gender, physician and hospital characteristics, demographic characteristics and co-morbid illnesses of patients, as well as in-hospital mortality. The Ontario Registered Persons Database provided the 30-day and 1 year mortality, both in and out of hospital. Algorithms used to link data across databases have proven reliability and validity.

Administrative databases used do not contain personal income data of the individual patients. They do, however, include the Forward Sortation Area (FSA) (the first three digits of the postal code), which was used to impute the patient' s median neighborhood income from the 1996 Canadian Census. Of the 504 FSAs in Ontario, the median neighborhood income for 11 was suppressed by Statistics Canada due to small sample size.

### Statistical Analysis

Median neighborhood income was broken down into quintiles for analysis. Baseline data across socioeconomic quintiles were compared using the Cochrane-Mantel-Haenszel chi-square for the categorical data, and weighted linear regression for continuous data. Kaplan-Meier survival curves were created to illustrate 30-day and 1 year mortality of the cohort by income quintile. Cox proportional hazards and logistic regression was used to determine the relation of median neighbourhood income to 30-day and 1-year mortality, adjusting for potentially confounding variables known or suspected to influence mortality risk: age, gender, co-morbidity (Charlson index score ≥ 1), specialty of attending physician and hospital status (teaching or non-teaching).

All statistics was done using SAS software (version 8.2), survival curve graphs were done using Microsoft Excel (version 9.0.0.3822).

## Results

### Baseline Data

The cohort consisted of 61,086 people, of whom 60,457 could be assigned a SES quintile (99% of the cohort), and hence could be used in the analyses. Table 1 shows the baseline characteristics of the population. There was a significant difference (p < 0.0001) in co-morbidity among the classes. The higher social classes had the higher co-morbidity (Charlson score >1) of cancer and ischemic heart disease, but there was no difference in the prevalence of chronic lung disease, chronic renal failure or congestive heart failure. The higher social classes were admitted more often to teaching hospitals,(as compared to community hospitals) and were attended by specialists (internal medicine, respirology) more frequently than general practitioners (see Table 1). Table 2 indicates no difference was found in length of stay across the income quintiles. Persons from the higher income quintiles were more likely to be discharged home.

**Table 1:** Baseline Characteristics of Pneumonia Patients According to Neighbourhood Median Income

**Characteristics**	**Income Quintile**					**P-value**
	**1**	**2**	**3**	**4**	**5**	

	N = 15057	N = 14655	N = 12268	N = 10310	N = 8167	

Neighborhood Income ($)						0.0039
*Median*	16433	18572	20256	22982	26786	
*Interquartile Range*	15408–16988	18056–19201	20043–21020	22186–24258	25899–29710	
Mean Age (yr)	78.03+/- 7.65	77.94+/- 7.55	78.07+/- 7.59	78.29+/- 7.58	78.51+/- 7.72	0.0370
Female sex (%)	47.23	48.29	47.21	49.20	49.44	0.0007
Comorbid conditions (%)						
*Chronic Lung Disease*	6.91	7.55	6.33	7.59	6.61	0.5382
*Congestive Heart Failure*	4.30	4.79	4.73	4.76	3.87	0.4348
*Ischemic Heart Disease*	11.60	12.90	14.11	14.31	13.22	<0.0001
*Peripheral Vascular Disease*	1.34	1.30	1.36	1.26	1.04	0.1003
*Chronic Renal Failure*	0.90	0.87	1.04	1.09	0.73	0.9210
*Diabetes*	6.97	8.02	7.44	7.38	6.09	0.0199
*Cancer*	2.89	3.43	3.73	3.77	4.04	<0.0001
*Charlson score > 1 (%)*	28.76	28.98	30.91	31.74	31.50	<0.0001
*Specialty of Attending Physician (%):*						<0.0001
*General Practice*	54.78	62.27	56.26	43.96	42.07	
*Internal Medicine*	26.69	23.82	26.18	34.24	35.14	
*Respirology*	6.16	5.43	5.98	7.19	8.90	
*Other*	12.37	8.47	11.57	14.62	13.89	
Teaching Hospital (%)	17.63	11.31	17.12	23.66	23.58	<0.0001

**Table 2:** Pneumonia Treatment and Outcomes According to Quintile of Median Neighborhood Income

**Outcome/Treatment N (%)**	**Income Quintile**					**P-value**
	**1**	**2**	**3**	**4**	**5**	

	N = 15057	N = 14655	N = 12268	N = 10310	N = 8167	

Length of Stay						0.1448
*Mean +/- SD (days)*	9.41 +/- 13.0	8.95 +/- 12.5	9.54 +/- 13.9	9.59 +/- 14.0	9.96 +/- 14.0	
*Median (Interquartile Range)*	6 (4–10)	6 (4–10)	6 (4–10)	6 (4–10)	6 (4–11)	
Acute Length of Stay						0.2362
*Mean +/- SD (days)*	8.39 +/- 8.52	7.93 +/- 7.58	8.29 +/- 8.12	8.37 +/- 8.44	8.86 +/- 9.60	
*Median (Interquartile Range)*	6 (4–10)	6 (4–10)	6 (4–10)	6 (4–10)	6 (4–11)	
Discharge Destination						<0.0001
*Acute Care Hospital*	433 (2.88)	284 (1.94)	224 (1.83)	128 (1.24)	99 (1.21)	
*Chronic Care Hospital*	263 (1.75)	365 (2.49)	285 (2.32)	211 (2.05)	140 (1.71)	
*Rehabilitation Hospital*	57 (0.38)	106 (0.72)	92 (0.75)	102 (0.99)	93 (1.14)	
*Nursing Home*	361 (2.40)	289 (1.97)	229 (1.87)	187 (1.81)	174 (2.13)	
*Home Care Program*	2101 (13.95)	1890 (12.90)	1754 (14.30)	1561 (15.14)	1123 (13.75)	
*Home*	11648 (77.36)	11484 (78.36)	9511 (77.53)	7998 (77.58)	6455 (79.04)	

### Mortality

**Figure 1:**
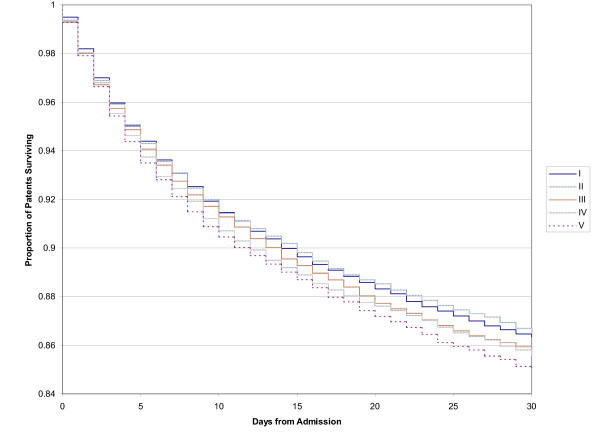
30 day mortality after initial admission for pneumonia, survival curves for social class quintiles from lowest (I) to highest (V)

**Figure 2:**
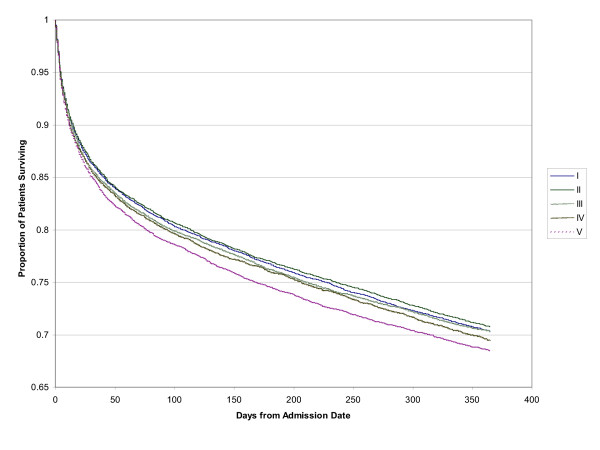
1 year mortality after initial admission for pneumonia, survival curves for social class quintiles from lowest (I) to highest (V).

The Kaplan-Meier survival curves for pneumonia mortality resulted in similar findings for both the 30-day mortality and the 1-year mortality (see Fig 1 and 2).

Multivariate modelling with logistic regression resulted in no significant difference in mortality (both 30-day and 1 year) across the income quintiles after adjustment for age, gender, co-morbidity (Charlson index score ≥ 1), specialty of attending physician and hospital teaching status (see Table 3 Cox models were completed, but the assumptions of the model violated. Odds Ratios were similar to the logistic models). The odds of death after 30-days for the quintiles compared to the lowest income quintile (quintile 1) were 1.02 (95% CI: 0.95–1.09) for quintile 2, 1.04 (95% CI: 0.97–1.12) for quintile 3, 1.01 (95% CI: 0.94–1.08) for quintile 4 and 1.03 (95% CI: 0.96–1.12) for the highest income quintile (quintile 5). The results were very similar for 1 year mortality, where, compared to the lowest income quintile the odds ratios were 1.01 (95% CI: 0.96–1.06) for quintile 2, 0.99 (95% CI: 0.94–1.04) for quintile 3, 0.99 (95% CI: 0.93–1.05) for quintile 4 and 1.03 (95% CI: 0.97–1.10) for the highest income quintile.

**Table 3:** Odds of Dying after Initial Admission for Pneumonia after 30 Days and 1 Year, Adjusted for Gender, Age, Specialty of Attending Physician, Hospital Teaching Status and Socio-economic Status

**Characteristic**	**Odds Ratio (65% CI)**	**P-value**
**30-Day Mortality**		

Characteristics of Patient		
*Female Sex*	0.78 (0.74–0.82)	<0.0001
*Charlson Index*	1.81 (1.71–1.91)	<0.0001
Age 65–74*	1.00	
*75–84*	1.55 (1.46–1.65)	<0.0001
*85–105*	3.02 (2.83–3.21)	<0.0001
Income Quintile 1*	1.00	
*2*	1.02 (0.95–1.09)	0.64
*3*	1.04 (0.97–1.12)	0.23
*4*	1.01 (0.94–1.08)	0.86
*5*	1.03 (0.96–1.12)	0.41
Characteristics of Hospital		
*Teaching Hospital (vs. non)*	0.88 (0.83–0.94)	0.0003
Specialty of Attending Physician		
*General Practitioner*	0.65 (0.60–0.70)	<0.0001
*Internal Medicine*	0.93 (0.87–1.00)	0.073
*Respirology*	0.84 (0.75–0.94)	0.0016
Other*	1.00	
**1-Year Mortality**		
Characteristics of Patient		
*Female Sex*	0.68 (0.65–0.70)	<0.0001
*Charlson Index*	2.09 (2.01–2.18)	<0.0001
Age 65–74*	1.00	
*75–84*	1.45 (1.39–1.52)	<0.0001
*85–105*	2.86 (2.73–3.01)	<0.0001
Income Quintile 1*	1.00	
*2*	1.01 (0.96–1.06)	0.72
*3*	0.99 (0.94–1.04)	0.73
*4*	0.99 (0.93–1.05)	0.70
*5*	1.03 (0.97–1.10)	0.31
Characteristics of Hospital		
*Teaching Hospital (vs. non)*	1.01 (0.96–1.06)	0.67
Specialty of Attending Physician		
*General Practitioner*	0.67 (0.63–0.71)	<0.0001
*Internal Medicine*	0.82 (0.77–0.87)	<0.0001
*Respirology*	0.78 (0.72–0.86)	<0.0001
Other*	1.00	

(* = reference group)

Women had lower odds of dying for both 30-day and 1-year mortality respectively (OR = 0.780, 95% CI: 0.744–0.818; OR = 0.68, 95% CI: 0.65–0.70) than men. The middle and oldest age groups (75–84, 85+) had higher odds of dying than the lowest age group (65–74) (30-day mortality: OR = 1.55, 95% CI: 1.47, 1.65; OR = 3.017, 95% CI: 2.83, 3.21; 1-year mortality: OR = 1.55, 95% CI: 1.39, 1.52; OR = 2.866, 65% CI: 2.73–3.01). The presence of another illness (Charlson co-morbidity index > = 1) significantly increased the mortality (OR = 1.81, 95% CI: 1.72, 1.91; OR = 2.094, 95% CI: 2.01–2.18). The specialty of the attending physician was also significant: compared to other types of physicians, treatment by a general practitioner had the highest protective effect on 30-day mortality (OR = 0.65, 95%CI = 0.599–0.696). Respirologist care was protective (OR = 0.83, 95% CI = 0.75, 0.94), while internal medicine practitioners did not have significant protective effects. One-year mortality was significantly affected by all three physician specialty groups studied; compared to other types of physicians, treatment by a general practitioner had the highest protective effect (OR = 0.67, 95% CI = 0.63–0.71), then respirologist (OR = 0.79, 95% CI = 0.72–0.86), then internal medicine practitioners (OR = 0.82, 95% CI: 0.77–0.87). Hospital teaching status was significant for 30-day mortality (OR = 0.89, 95% CI: 0.83–0.95) but not for 1-year mortality.

## Discussion

There is increasing evidence from diverse observational studies that low SES is associated with adverse health outcomes [11]. The findings of this study do not support the existence of an association between socioeconomic status and mortality subsequent to CAP. The study results indicate that community acquired pneumonia is a condition associated with high mortality, and that gender, age and co-morbidity most significantly influence outcome. The study results are similar to a recent study by Kaplan et al. reporting a 33.6% mortality rate in survivors of CAP [12]. The results underscore the high prevalence, resource intensity and mortality associated with CAP, particularly in older persons [1].

The strengths of this study are its population base, large sample size, accurate linkages and detailed follow up. The study captured all pneumonia admissions for those over 65 in the province of Ontario. The pneumonia diagnostic codes (ICD-9 codes 480–487) were the same as in previous studies [13-15].

The study is limited by the use of proxy measures for socioeconomic status, namely income data from median neighborhood income. Currently there is considerable debate as to the proper measure of SES and controversy as to whether area level measures are valid for imputing SES. The measure of SES status employed in this study is identical to that used in other published studies demonstrating significant associations with mortality and access to healthcare services for myocardial infarction and stroke[5,6]. The use of area-level information applying to individuals forces consideration of the ecological fallacy. However, others have argued for the validity of using income quintiles as a proxy for socioeconomic status [16-19]. Mustard found that ecologic measures of income are highly correlated to individual income. Hence use of such proxy measures is justified when individual level data is not available [20].

There have been conflicting results concerning the relationship between socioeconomic status and pneumonia (both diagnosis and outcome). Wood [13] found an increased relative risk (RR 2.3 95% CI: 1.4–4.0) for lower social class quintiles and pneumonia and bronchitis mortality. Stelianides found that the duration of hospitalization was 5.9 days longer for low SES patients as compared to high SES patients (p < 0.003), but found no differences in mortality or ICU admission [14]. Singh and Siahpush [15] found a relative risk of 2.69 (p < 0.05) for the lowest compared to the highest income group with pneumonia and influenza mortality. Other studies, looking at pneumonia diagnosis and SES found no relation between the two [21,22]. Our study found differences in the process of care, in that higher SES patients were more likely to be treated by specialists and in academic teaching centres, but not in outcomes, as mortality and length of stay were not significantly different between SES levels. Interestingly, as a secondary outcome, those with family physicians had lower mortality than those without, suggesting that provision of primary care has a protective effect. This finding bears further exploration. As well, as in other studies [5,6], academic health sciences centres had better mortality outcomes for acute care.

The relation between SES and health is not completely understood, but theories abound. Among the explanations for the relation found between disease outcomes and socioeconomic status relates to equitable access to health services as well as more negative lifestyle and environmental exposures (higher rates of smoking, worse air quality). How can we explain that our data does not corroborate past findings or theories? One possible explanation may lie in the nature of the management of pneumonia. Myocardial infraction and stroke, where differences in outcomes and SES in this population have been reported, increasingly rely on the provision of timely and specialized technology, diagnosis and management. Management of pneumonia is, for the most part, a relatively low technology process. The majority of patients were cared for by primary care providers. Hence access to care seems relatively unproblematic in this cohort. However, pneumonia remains, as it was in Osler's day, a potent force of mortality and socioeconomic status provides no advantage or protection.

## Conclusion

In this population based, retrospective cohort study of older persons in the province of Ontario, socioeconomic status was not a factor in increasing the risk of death subsequent to hospital admission for community acquired pneumonia. Male gender, age and co-morbid illness significantly increase both 30 day and one year mortality. Female gender is associated with significantly reduced risk. Having a primary care provider and being cared for in an academic health sciences centre also reduced the mortality risk.

## Competing Interests

The author(s) declare that they have no competing interests.

## Authors' Contributions

RU initiated the idea for the study. LJ and MM co-wrote the grant with RU. LV wrote the first draft of the article and carried out the statistical analysis. RM provided intellectual input to the study design and analysis. All contributed intellectual input into the study. All participated in the revision of drafts and approve of the final draft.
